# Targeted Next-Generation Sequencing Reveals Hot Spots and Doubly Heterozygous Mutations in Chinese Patients with Familial Cardiomyopathy

**DOI:** 10.1155/2015/561819

**Published:** 2015-06-24

**Authors:** Yue Zhao, Yue Feng, Yun-Mei Zhang, Xiao-Xue Ding, Yu-Zhu Song, A-Mei Zhang, Li Liu, Hong Zhang, Jia-Huan Ding, Xue-Shan Xia

**Affiliations:** ^1^Faculty of Life Science and Technology, Center for Molecular Diagnosis in Yunnan Province, Kunming University of Science and Technology, Kunming 650500, China; ^2^The Cardiac Department, The First Hospital of Yunnan Province, Kunming 650034, China

## Abstract

As a common cardiac disease mainly caused by gene mutations in sarcomeric cytoskeletal, calcium-handling, nuclear envelope, desmosomal, and transcription factor genes, inherited cardiomyopathy is becoming one of the major etiological factors of sudden cardiac death (SCD) and heart failure (HF). This disease is characterized by remarkable genetic heterogeneity, which makes it difficult to screen for pathogenic mutations using Sanger sequencing. In the present study, three probands, one with familial hypertrophic cardiomyopathy (FHCM) and two with familial dilated cardiomyopathy (FDCM), were recruited together with their respective family members. Using next-generation sequencing technology (NGS), 24 genes frequently known to be related to inherited cardiomyopathy were screened. Two hot spots (TNNI3-p.Arg145Gly, and LMNA-p.Arg190Trp) and double (LMNA-p.Arg190Trp plus MYH7-p.Arg1045His) heterozygous mutations were found to be highly correlated with familial cardiomyopathy. FDCM patients with doubly heterozygous mutations show a notably severe phenotype as we could confirm in our study; this indicates that the double mutations had a dose effect. In addition, it is proposed that genetic testing using NGS technology can be used as a cost-effective screening tool and help guide the treatment of patients with familial cardiomyopathy particularly regarding the risk of family members who are clinically asymptomatic.

## 1. Introduction

Inherited cardiomyopathy is a chronic myocardial disorder affecting all ethnic groups, resulting in a large cost burden to social health systems. The World Health Organization (WHO) has divided cardiomyopathy into four categories: hypertrophic cardiomyopathy (HCM), dilated cardiomyopathy (DCM), arrhythmogenic right ventricular cardiomyopathy (ARVC), and restrictive cardiomyopathy (RCM) [1]. Among these disorders, HCM and DCM occur most frequently in the Chinese population.

HCM is a frequent genetic heart disease with autosomal dominant inheritance and is one of the most common causes of sudden cardiac death (SCD), especially in adolescents and young athletes [2, 3]. Clinically, HCM is characterized by unexplained left ventricular hypertrophy, myocyte disarray, and myocardial fibrosis [4]. The incidence of HCM was estimated to be at least 1/500 individuals [5]; thus, approximately 2 million patients in China are affected by HCM. The first gene mutation identified to be causing heart disease was reported in 1993 [6]; since then, more than 1400 mutations have been detected in at least 20 genes [7] that encode sarcomere proteins, calcium-handling proteins, Z-disc proteins, and so forth. In particular, MYH7 and MYBPC3 sarcomere proteins mutations are most commonly related to HCM [8].

Another common inherited cardiomyopathy, DCM, is clinically characterized by left ventricular dilation with systolic dysfunction and affects at least 1/2500 of the general population worldwide [9]. Pathogenic gene mutations play an important role in DCM pathogenesis and mostly follow Mendelian autosomal dominant patterns; however, recessive as well as X-linked inheritance may also be involved [10]. About 20~48% of DCM patients have familial forms of the disease, called familial DCM (FDCM). FDCM is often characterized by genetic heterogeneity and shows high variability even between family members; the age of onset and disease progression also differ considerably between individuals with FDCM. To date, mutations in more than 50 genes have been reported to cause FDCM, including mutations in genes encoding sarcomere proteins, cytoskeletal proteins, and nuclear envelope proteins [10].

Genetic testing may provide the genetic information required for clinical diagnosis of inherited cardiomyopathy. Once a disease-related mutation is identified in a proband, testing for this mutation should be conducted on other family members to predict the disease risk and guide preventative measures and treatment [11, 12]. In this study, comprehensive genetic analysis of 24 genes frequently known to be related to inherited cardiomyopathy (*ABCC9*,* CAV3*,* DES*,* MYBPC3*,* MYL2*,* PRKAG2*,* PSEN1*,* PSEN2*,* SGCD*,* TNNC1*,* TPM1*,* MYH6*,* MYH7*,* TNNT2*,* SCN5A*,* TNNI3*,* MYL3*,* MYPN*,* LAMA4*,* RBM20*,* VCL*,* LDB3*,* ACTN2*, and* LMNA*) was performed in FHCM and FDCM patients by using next-generation DNA sequencing (NGS) technologies to identify their potential pathogenic mutations, as well as to determine their value in clinical diagnosis and in guiding family therapy management.

## 2. Materials and Methods

### 2.1. Patient Data

In this study, the patient from Family A was diagnosed with FHCM based on the American College of Cardiology Foundation/American Heart Association (ACCF/AHA) criteria [4], using the following inclusion criterion: wall thickness >15 mm on echocardiography, with wall thickness of 13-14 mm being considered borderline. The two patients in Family B had been previously diagnosed with FDCM in accordance with European guidelines [13]. The patients resided in Yu Xi, in the central region of the Yunnan province, China. Their demographic and clinical information, including family history, clinical symptoms, echocardiography results, and 12-lead electrocardiography (ECG) records, were collected. Genetic testing was performed on the patients and their family members. This investigation was approved by the Institutional Ethics Committee of the First People's Hospital of Yunnan Province (Affiliated Hospital of Kunming University of Science and Technology), China.

### 2.2. Candidate Gene Sequencing

For each sample, genomic DNA was extracted from anticoagulated whole blood by using a commercial genomic DNA midiprep kit (AxyPrep, Corning, CA, USA). The PCR primer panel was designed to amplify the coding exons and partial introns of a number of genes frequently known to be related to inherited cardiomyopathy:* ABCC9*,* CAV3*,* DES*,* MYBPC3*,* MYL2*,* PRKAG2*,* PSEN1*,* PSEN2*,* SGCD*,* TNNC1*,* TPM1*,* MYH6*,* MYH7*,* TNNT2*,* SCN5A*,* TNNI3*,* MYL3*,* MYPN*,* LAMA4*,* RBM20*,* VCL*,* LDB3*,* ACTN2*, and* LMNA*. The amplicons were submitted to Agilent Technology (CA, USA) for construction of a multitarget gene library and then sequenced using a Genome Analyzer IIx (Illumina, CA, USA).

### 2.3. Molecular Genetic Analysis

The sequencing results were aligned to the National Center for Biotechnology Information (NCBI) human reference genome assembly (GRCh37/hg19), and PCR duplicates were removed using the SAM tools software package (version 0.1.16) [14]. To identify nucleotide mutations, including both unknown mutations and those that have been reported in the dbSNP137 (http://www.ncbi.nlm.nih.gov/projects/SNP) and 1000 Genome Project (http://www.1000genomes.org), the genomic variations in the sequences were annotated with the ANNOVAR software [15]. Synonymous and noncoding region mutations were excluded in this step, and the remaining coding region variations were considered putative pathogenic mutations. Putative pathogenic mutations were analysed computationally using the PolyPhen-2, SIFT, and MutationTaster [16–18] algorithms. These algorithms can distinguish mutations with functional effects from other neutral mutations.

### 2.4. Mutation Validation

The putatively pathogenic mutations were verified using custom-designed Sanger sequencing methods. Exon 7 of* TNNI3*, exon 3 of* LMNA*, and exon 25 of* MYH7* were amplified using primers covering exon 7 of the mutated* TNNI3*-c.433C>G, primers covering exon 3 of the mutated* LMNA*-c.568C>T, and primers covering exon 25 of the mutated* MYH7*-c.3134G>A, respectively (Table 2). Genomic sequences of the family members of the patients were also confirmed using the Sanger sequencing platform after PCR amplification of the corresponding exons to confirm the results.

## 3. Results

### 3.1. Familial Phenotypes of HCM and DCM

**Figure 1:**
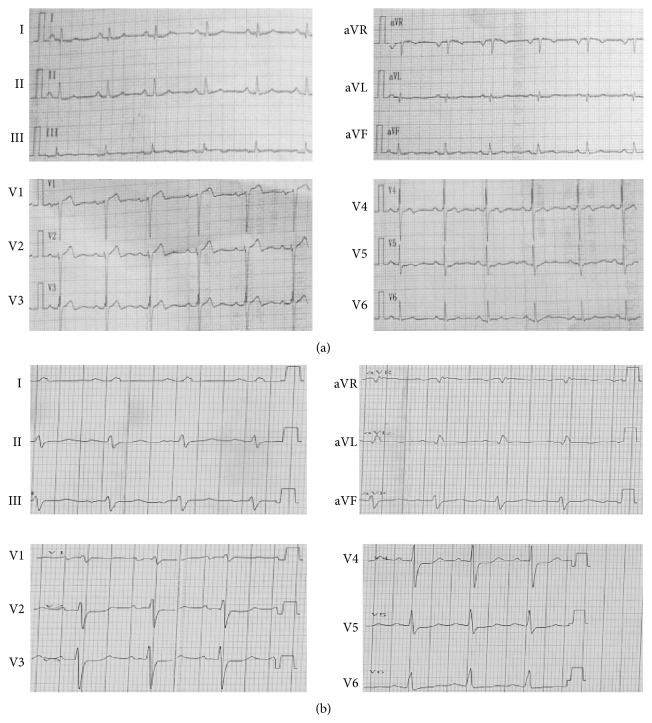
The electrocardiogram of probands with familial hypertrophic cardiomyopathy and familial dilated cardiomyopathy. (a) shows the electrocardiogram of the proband with HCM from family A (II: 3); the Q-wave was abnormal in sidewalls and the T-wave is changed; (b) shows the electrocardiogram of the proband with DCM from family B (II: 3); it reveals a significant ST-segment depression.

**Figure 2:**
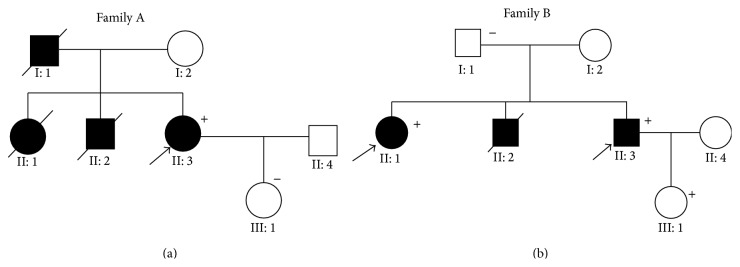
The pedigrees of the families with hypertrophic and dilated cardiomyopathy. Male family members are indicated by squares; female family members are indicated by circles, deceased individuals are indicated by symbols with a strikethrough, the unaffected individuals are represented by open symbols, and the solid symbols represent affected individuals. In addition, the probands are marked with a black arrow. The presence of a mutation was indicated by a “+” sign and the absence of mutations was indicated by a “–” sign. Family A: II: 3 is the proband, I: 1 and II: 2 died of sudden cardiac death, and III: 1 is clinically unaffected; the other clinical data were unavailable; Family B: II: 1 and II: 3 are the probands, II: 2 died of sudden cardiac death, and III: 1 mutation is present, but individual is clinically unaffected.

Families A and B were diagnosed with HCM and DCM, respectively; data regarding their clinical symptoms and the demographic information were collected during interviews. In family A, the proband II: 3 was a 44-year-old woman with clinical symptoms of dyspnoea and chest tightness after exercise. Her father (I: 1), sister (II: 1), and brother (II: 2) died of SCD around the age of 40 years. Her daughter (III: 1, 22 years of age) presented as clinically unaffected at the time of the interview (Figure 2(a)). No clinical data were available for III: 1. According to the clinical records of this proband (II: 3), her heart rate was 68 beats/min, and the ECGs showed changes in T-waves, which is common in patients with HCM. Additionally, the Q-wave was abnormal in the sidewalls, while the QRS (86 ms) duration was within the normal range (Figure 1(a)). Moreover, echocardiography showed an increase in the interventricular septum thickness (IVST, 14.8 mm), as well as in the thickening of the left atrium (LA, 32 mm).

In family B, the proband II: 3 was a 43-year-old man with severe DCM symptoms. His elder brother (II: 2) had died of SCD, and his sister (II: 1) had been diagnosed with DCM before that, while his daughter (III: 1) and father (I: 1) showed no clinical symptoms. No data were available for the individuals Family B III: 1 and B I: 1 (Figure 2(b)). Echocardiographic tests for II: 3 showed severe cardiac enlargement and increased left ventricular end-diastolic diameter (LVEDD, 77.8 mm), while the left ventricular end-systolic diameter (LVESD, 69.8 mm) and left ventricular ejection fraction (LVEF, 21% < 50%) were lesser than those observed in the general population. His heart rate was 78 beats/min, and ECG revealed significant ST-segment depression in II, III, aVF, V2, V3, and V4, which is indicative of ischemia, a predisposing condition seen in patients with FDCM [19]. Notably, a widened QRS complex (120 ms > 110 ms) (Figure 1(b)) may indicate a bundle branch block.

### 3.2. Summary of Targeted Sequencing for the Patients and Their Families

We analysed the number of reads that covered the variations in 24 genes frequently known to be related to inherited cardiomyopathy; we considered that a read depth ≥30 reads (30x, Q30 = 99.9% chance of the correct base being called) for each targeted variation indicated that it was correctly covered [20]. A summary of the nonsynonymous mutations and coverage of three patients (Family A, II: 3; Family B, II: 1 and II: 3), after exclusion of synonymous and noncoding region mutations, is shown in Table 1. Using bioinformatics analysis of the sequencing results, we identified the pathogenic mutations TNNI3-p.Arg145Gly in Family A and LMNA-p.Arg190Trp plus MYH7-p.Arg1045His in Family B. The functional impact of amino acid changes was predicted using three computational programs (PolyPhen-2, SIFT, and MutationTaster). The mutations TNNI3-p.Arg145Gly, LMNA-p.Arg190Trp, and MYH7-p.Arg1045His change the amino acid sequence and may have some impact on the function of coding proteins.

### 3.3. Mutations in* TNNI3*,* LMNA*, and* MYH7*

**Figure 3:**
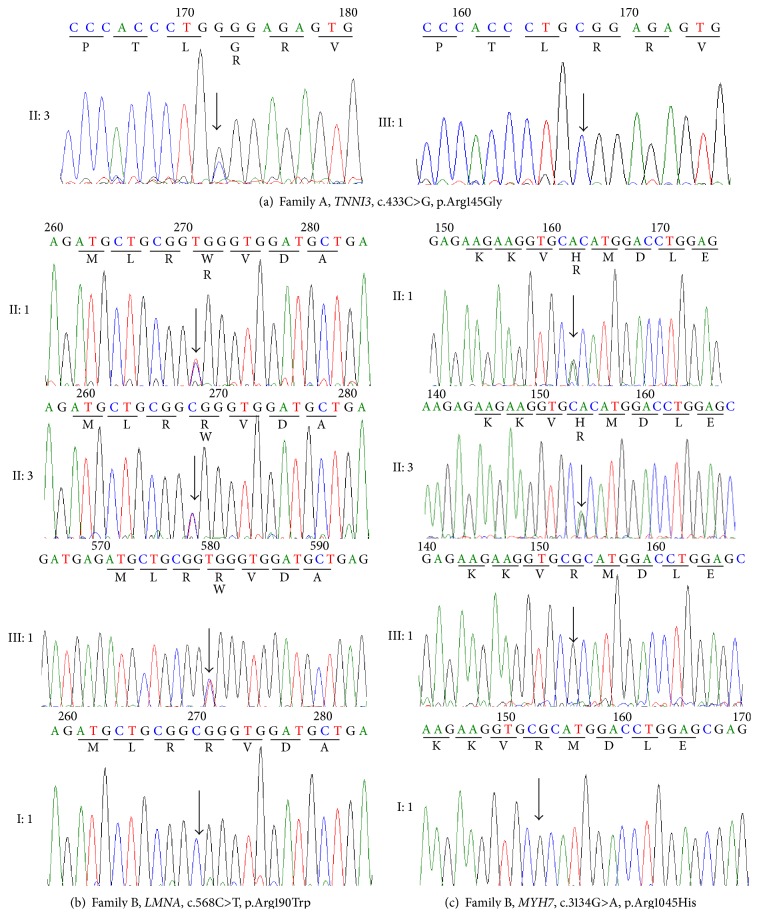
Results of the Sanger sequencing analysis. (a) shows the results for the TNNI3-p.Arg145Gly mutation in Family A; the results were positive in II: 3 and negative in III: 1; (b) shows the results for the LMNA-p.Arg190Trp mutation in Family B; the results were positive in II: 1, II: 3, and III: 1 and negative in I: 1; (c) shows the results for the MYH7-p.Arg1045His mutation in Family B; family members II: 1 and II: 3 tested positive, and III: 1 and I: 1 tested negative.

In family A, the proband (II: 3) was diagnosed with typical HCM. In the sequenced genomic regions, we identified the known C>T pathogenic heterozygotic mutation located at nucleotide position c.433 (Transcript name: NM_000363.4) in* TNNI3* exon 7 (Table 1 and Figure 3(a)). This mutation results in the replacement of arginine at the 145th amino acid position by glycine (p.Arg145Gly). Using Sanger sequencing, this mutation was verified; it was not found in the daughter (Figure 3(a)). Further analysis showed that the amino acid Arg145 is highly conserved in many species and is localized in the first actin binding domain of the TNNI3 protein.

Molecular genetics analysis for family B showed that the probands II: 1 and II: 3 have the pathogenic doubly heterozygous mutations c.568C>T within exon 3 of* LMNA* and c.3134G>A within exon 25 of* MYH7* (Table 1 and Figures 3(b) and 3(c)). These mutations are predicted to cause substitution of a nonsynonymous charged arginine with a tryptophan at codon 190 (Transcript name: NM_005572.3, p.Arg190Trp) and an arginine with a histidine at codon 1045 (Transcript name: NM_000257, p.Arg1045His). Although the father (I: 1) and daughter (III: 1) of the proband II: 3 did not have clinical symptoms (e.g., tightness, dyspnoea, and dizziness), the same pathogenic mutation LMNA-p.Arg190Trp was also identified in the daughter (III: 1) by Sanger sequencing. In contrast, this mutation (LMNA-p.Arg190Trp) was not found in the father (I: 1) (Figure 3(b)). The identified mutated site of LMNA is also highly conserved among species and is localized in the Coil1b region of the LMNA protein. Interestingly, we also found a second pathogenic mutation (MYH7-p.Arg1045His) in family B in the probands II: 1 and II: 3, in addition to the LMNA-p.Arg190Trp mutation. We found that the amino acid MYH7-p.Arg1045 is highly conserved in many species.

## 4. Discussion

In this study, candidate genes were sequenced in patients with familial cardiomyopathy by using NGS technologies. Many nonsynonymous variations were detected in the familial cardiomyopathy patients and per nonsynonymous variation in coverage of more than 30x (Table 1), including three pathogenic heterozygotic mutations (*TNNI3*, c.433C>G, p.Arg145Gly;* LMNA*, c.568C>T, p.Arg190Trp; and* MYH7*, c.3134G>A, p.Arg1045His). Further, PCR and Sanger sequencing were used to verify those three mutations.

The* TNNI3* gene is located on chromosome 19q13.4 and encodes cardiac troponin I type 3. Together with the TNNT2 and TNNC1 subunits, TNNI3 forms the heterotrimeric troponin complex in the thin filaments of the cardiac striated muscle, and it is a key regulatory protein of the thin filament [21, 22]. TNNI3 contains five main functional regions: a region containing phosphorylation sites, a TNNC1 binding domain, a TNNT2 binding domain, the first actin binding domain, and the second actin binding domain (Table 3). It is the main inhibitory subunit of the troponin complex, and after calcium binding to troponin C, it blocks interaction between myosin and actin via tropomyosin and indirectly inhibits actomyosin ATPase activity. Mutation of the* TNNI3* gene and the subsequent alterations in the protein may interfere with its binding to the other subunits and disrupt the function of the entire troponin complex. Furthermore, the relevant evidence indicates that the mutated TNNI3 protein (p.Arg145Gly) increased activity of actomyosin ATPase activity via increasing the sensitivity of Ca^2+^ [23]. This causes increased Ca^2+^ sensitivity in cardiac myofilaments, which results in increased activity of the sarcomere proteins, eventually leading to the occurrence of HCM. The prevalence of* TNNI3* mutations is approximately 5% in HCM. To date, approximately 30 mutations have been identified to be associated with HCM (Table 3), and the most frequent* TNNI3* mutations have been reported in exons 7 and 8, which encode the domains interacting with cardiac troponin C (TNNC1) and cardiac actin (ACTC1) (Table 3). The TNNI3-p.Arg145Gly mutation identified here has been previously described in American and Korean patients (Table 3). However, to our knowledge, our study is the first report of the TNNI3-p.Arg145Gly mutation in a Chinese patient with FHCM. It is worth noting that the p.Arg145Gly mutation is a hot spot of TNNI3 and is highly correlated with HCM in American, Korean, and Chinese populations (Table 3).

The clinical diagnosis of HCM is based on the criterion of maximal wall thickness greater than or equal to 15 mm; our patient (Family A, II: 3) had wall thickness (14.8 mm) close to 15 mm. However, studies on genotype-phenotype correlations have verified that essentially any wall thickness (including normal wall thickness) is compatible with the presence of a HCM mutant gene but also have a high incidence of sudden death [24, 25]. Our interpretation is that mutation (p.Arg145Gly) in the gene encoding TNNI3 is delayed or subclinical cardiac hypertrophy until middle age or old age; this result indicates that genetic testing has an important diagnostic value, especially for clinically equivocal patient.

The* LMNA* gene is located on chromosome 1q22 and encodes two major isoforms of lamin A/C, which is the main nuclear protein component in mammals and acts as a meshwork structure. The LMNA protein contains four major coiled-coil domains (Coil1a, Coil1b, Coil2a, and Coil2b) and three insertion regions (L1, L2, and L12) [26]. Generally, the* LMNA* gene is highly conserved throughout evolution. However, approximately 40 disease-causing mutations have been described in different populations (Table 4).* LMNA* may be one of the most frequent disease-associated genes for FDCM and has been shown to be associated with a severe clinical phenotype; the prevalence of mutations in* LMNA* is approximately 10% in DCM patients [27]. The documented data (Table 4) suggest that mutations in DCM may occur almost anywhere in* LMNA*; however, the Coil1b region, which is important for lamin A/C dimerization and lamin B interaction, seems to be affected most frequently. The LMNA-p.Arg190Trp mutation has also been documented in previous studies [28–34] (Table 4), but, to our knowledge, this is the first report of the LMNA-p.Arg190Trp mutation in a case of Chinese familial DCM. This finding further illustrates that the LMNA-p.Arg190Trp mutation is a hot spot in familial DCM in Chinese patients as well as in the general population. Interestingly, this pathogenic mutation (LMNA-p.Arg190Trp) was also identified in the proband II: 3's daughter (III: 1) who showed no clinical symptoms; it illustrates that III: 1 has an onset of the disease that appears delayed. Based on these findings, regular clinical cardiovascular testing and further genetic screening are recommended for her and all first-degree relatives of family B for future clinical management of this case of familial cardiomyopathy.

An additional mutation (MYH7-p.Arg1045His) was found in family B in patients II: 1 and II: 3; the proband (family B, II: 3) with the doubly heterozygous mutations displayed a more malignant clinical phenotype of cardiomyopathy (Figures 2(b) and 3(c)). This described for the first time the double heterozygous mutations of LMNA-p.Arg190Trp plus MYH7-p.Arg1045His in FDCM patients. The* MYH7* gene, which encodes the myosin heavy chain, is one of the most important causative genes in inherited cardiomyopathy. Previous studies have reported a relationship between the MYH7-p.Arg1045His mutation and HCM [35, 36]; however, our study is the first to discover the association of the MYH7-p.Arg1045His mutation with FDCM. We believe that these results indicate that double mutations (LMNA-p.Arg190Trp plus MYH7-p.Arg1045His) had a dose effect. As a result, it led to the patients (LMNA-p.Arg190Trp plus MYH7-p.Arg1045His carriers) showing a clinical malignant phenotype. This result is an additional indication of the genetic heterogeneity of inherited cardiomyopathy.

## 5. Study Limitations

In this study, the number of recruited participants was limited, and the obtained clinical data were not consistent for all subjects. In addition, we used the accepted normal range of each clinical parameter in this study. A larger study on Chinese familial cardiomyopathies will be essential to confirm further the mutations identified in this study.

## 6. Conclusions

To our knowledge, this is the first study to discover the mutations TNNI3-p.Arg145Gly and LMNA-p.Arg190Trp in Chinese patients with familial HCM and DCM, respectively. A double mutation (LMNA-p.Arg190Trp plus MYH7-p.Arg1045His) was first discovered in familial DCM. These mutations were identified using NGS technologies and were confirmed by Sanger sequencing. Considering both previous data and our new findings, it is proposed that TNNI3-p.Arg145Gly and LMNA-p.Arg190Trp are hot spots of FHCM and FDCM, respectively. The discovery of the mutational status with respect to the double mutations (LMNA-p.Arg190Trp plus MYH7-p.Arg1045His) may be useful in family B for assessment of individuals at risk for familial DCM; the results further indicate the genetic heterogeneity of inherited cardiomyopathy. Genetic testing may provide more predictive information for inherited cardiomyopathy diagnosis, particularly regarding the risk to family members who are clinically asymptomatic. In addition, genetic testing of candidate genes by using NGS technologies is becoming increasingly viable and economical. In the future, NGS could be used as a complementary approach for the clinical diagnosis of FHCM and FDCM.

## Figures and Tables

**Table 1:** Potential of the nonsynonymous variations in patients with FHCM and FDCM.

Proband	Gene name	Transcript name	Coverage	Zygosity	Nucleic acid change	Amino acid change	rs ID
Family A II: 3	*LAMA4 *	NM_002290	184	Het	c.1471T>C	p.Tyr491His	rs1050348
	*TNNI3 *	NM_000363	111	Het	c.433C>G	p.Arg145Gly	rs104894724
	*MYPN *	NM_032578	169	Het	c.2072G>C	p.Ser691Ile	rs10997975
	*RBM20 *	NM_001134363	123	Hom	c.2303G>C	p.Trp768Ser	rs1417635
	*VCL *	NM_014000	172	Het	c.2801C>T	p.Ala934Val	rs16931179
	*SCN5A *	NM_198056	150	Het	c.1673A>G	p.His558Arg	rs1805124
	*LAMA4 *	NM_002290	235	Hom	c.3328G>A	p.Gly1110Ser	rs2032567
	*LDB3 *	NM_007078	192	Het	c.163G>A	p.Val55Ile	rs3740343
	*MYPN *	NM_032578	119	Het	c.2409C>G	p.Ser803Arg	rs3814182
	*ACTN2 *	NM_001103	186	Het	c.1423G>A	p.Asp475Asn	rs80257412
	*RBM20 *	NM_001134363	153	Het	c.3667G>C	p.Glu1223Gln	rs942077
	*MYL3 *	NM_000258	201	Het	c.92G>A	p.Arg31His	rs199639940

Family B II: 1	*LAMA4 *	NM_002290	191	Het	c.1471T>C	p.Tyr491His	rs1050348
	*RBM20 *	NM_001134363	116	Hom	c.2303G>C	p.Trp768Ser	rs1417635
	*MYH7 *	NM_000257	176	Het	c.3134G>A	p.Arg1045His	NA
	*VCL *	NM_014000	143	Het	c.2801C>T	p.Ala934Val	rs16931179
	*SCN5A *	NM_198056	133	Het	c.1673A>G	p.His558Arg	rs1805124
	*LAMA4 *	NM_002290	225	Hom	c.3328G>A	p.Gly1110Ser	rs2032567
	*MYH6 *	NM_002471	107	Het	c.3388G>A	p.Ala1130Thr	rs28730771
	*MYH6 *	NM_002471	158	Het	c.3302T>C	p.Val1101Ala	rs365990
	*TNNT2 *	NM_001001432	154	Het	c.740A>G	p.Lys247Arg	rs3730238
	*LMNA *	NM_170708	130	Het	c.568C>T	p.Arg190Trp	rs59026483
	*RBM20 *	NM_001134363	147	Het	c.3667G>C	p.Glu1223Gln	rs942077

Family B II: 3	*LAMA4 *	NM_002290	225	Het	c.1471T>C	p.Tyr491His	rs1050348
	*MYPN *	NM_032578	177	Het	c.1471T>C	p.Tyr491His	rs10823148
	*MYPN *	NM_032578	179	Het	c.2072G>A	p.Ser691Ile	rs10997975
	*RBM20 *	NM_001134363	125	Hom	c.2303G>C	p.Trp768Ser	rs1417635
	*SCN5A *	NM_198056	140	Het	c.1673A>G	p.His558Arg	rs1805124
	*LAMA4 *	NM_002290	286	Hom	c.3328G>A	p.Gly1110Ser	rs2032567
	*MYH6 *	NM_002471	121	Het	c.3388G>A	p.Ala1130Thr	rs28730771
	*MYH6 *	NM_002471	143	Het	c.3302T>C	p.Val1101Ala	rs365990
	*MYPN *	NM_032578	198	Het	c.2409C>G	p.Ser803Arg	rs3814182
	*LMNA *	NM_170708	114	Het	c.568C>T	p.Arg190Trp	rs59026483
	*MYPN *	NM_032578	199	Het	c.3403C>A	p.Pro1135Thr	rs7079481
	*MYPN *	NM_032578	186	Het	c.2120G>A	p.Ser707Asn	rs7916821
	*RBM20 *	NM_001134363	173	Hom	c.3667G>C	p.Glu1223Gln	rs942077
	*MYH7 *	NM_000257	169	Het	c.3134G>A	p.Arg1045His	NA
	*RBM20 *	NM_001134363	166	Het	c.3170G>A	p.Arg1057Gln	rs188054898

Het: heterozygotes; Hom: homozygous; NA: not applicable.

**Table 2:** PCR primers for amplification of the mutation sites.

Gene symbol	Nucleotide change	Exon	Primer (5′-3′)	Tm (°C)	Fragment size (bp)
*TNNI3 *	c.433C>G	7	Sense: GCCTAAGCCGGGAAGAGACTGGTA	55	437
			Antisense: GAGGACCCCTTACTAGCTGCTTCT		

*LMNA *	c.568C>T	3	Sense: GAGTAGCTGGGACTACAGGCGTGT	57	1338
			Antisense: ATCTGACTCCACATCCTGCGACC		

*MYH7 *	c.3134G>A	25	Sense: GGCAATCTCACAGTCCCCTAATAA	55	508
			Antisense: TTTTTGCCAGGGAGGACCATCTAA		

**Table 3:** The documented TNNI3 mutations in HCM.

Exon	Amino acid change	Local structure	Reported times	Population report group
3	p.Arg21Cys	TNNC binding domain	1	American [37] and Norwegian [35]
3	p.Arg13Cys	TNNC binding domain	1	Chinese [38]
4	p.Lys36Gln	TNNC binding domain	1	English [39]
5	p.Pro82Ser	TNNT2 binding domain	1	American [40]
7	p.Arg141Gln	First actin binding domain	2	American [41] and French [27]
7	p.Arg145Gly	First actin binding domain	8	American [2], Korean [42, 43], and Chinese [**this study**]
7	p.Arg145Gln	First actin binding domain	1	Japanese [44]
7	p.Asn185Lys	TNNC binding domain	1	English [39]
7	p.Ala157Val	TNNC binding domain	3	French [27], Norwegian [35], and Dutch [45]
7	p.Arg162Pro	TNNC binding domain	1	French [27]
7	p.Arg162Trp	TNNC binding domain	1	Japanese [44]
7	p.Arg162Gln	TNNC binding domain	2	American [41] and English [46]
7	p.Ser166Phe	TNNC binding domain	5	American [41] and Dutch [45]
7	p.Arg170Gln	TNNC binding domain	1	English [47]
7	p.Ser166Phe	TNNC binding domain	1	German [48]
7	p.Lys164Thr	TNNC binding domain	1	Dutch [49]
7	p.Asp180Gly	TNNC binding domain	1	Dutch [49]
7	p.Lys178del	TNNC binding domain	1	Dutch [49]
8	p.Arg186Gln	TNNC binding domain	1	French [27] and English [46]
8	p.Asp196Asn	Second actin binding domain	3	French [27], American [50], and Norwegian [35]
8	p.Gly203Ser	Second actin binding domain	1	Japanese [44]
8	p.Met201Thr	Second actin binding domain	1	Dutch [49]
8	p.Arg204Cys	Second actin binding domain	1	American [51]
8	p.Lys206Gln	Second actin binding domain	1	Japanese [44]
8	p.Glu209Ala	Second actin binding domain	5	Dutch [49]
8	p.Ile195Met	Second actin binding domain	1	American [51]

**Table 4:** The documented LMNA mutations in DCM.

Exon	Amino acid change	Local structure	Reported times	Population report group
1	p.Lys97Glu	Coil1b	1	Italian [30]
1	p.Arg101Pro	Coil1b	1	American [31]
1	p.Glu111^*∗*^	Coil1b	1	Italian [30]
1	p.Arg89Leu	Coil1b	2	American [31, 52]
1	p.Leu85Arg	Coil1b	1	American [53]
1	p.Arg60Gly	Coil1b	1	American [53]
1	p.Arg89Leu	Coil1b	1	American [52]
1	p.Glu82Lys	Coil1b	1	Chinese [54]
2	p.Arg166Pro	Coil1b	1	American [31]
2	p.Glu161Lys	Coil1b	1	German [26]
3	p.Arg190Trp	Coil1b	7	Spanish [28], Italian [30], American [31], German [32], English [33], Finland [34], Korea [28], and Chinese [**this study**]
3	p.Arg189Trp	Coil1b	1	Italy [12]
3	p.Glu203Val	Coil1b	1	German [26]
3	p.Arg190Gln	Coil1b	2	German [26] and American [31]
3	p.Glu203Lys	Coil1b	1	American [31]
3	p.Ile210Ser	Coil1b	1	American [31]
3	p.Asn195Lys	Coil1b	1	Dutch [55]
3	p.Glu203Gly	Coil1b	1	American [53]
3	p.Asn195Lys	Coil1b	1	American [53]
3	p.Asp192Gly	Coil1b	1	English [33]
4	p.Gly232Val	L12	1	Chinese (Taipei) [56]
4	p.Lys219Thr	L12	1	German [26]
4	p.Leu215Pro	L12	1	American [31]
4	p.Lys219Thr	L12	1	Italy [57]
4	p.His222Pro	L12	1	French [45]
4	p.Arg225^*∗*^	L12	2	American [31] and Dutch [55]
4	p.Gln234^*∗*^	L12	1	American [31]
6	p.Arg349Leu	Coil2b	1	Spanish [28]
6	p.Glu317Lys	Coil2b	1	Italian [30]
6	p.Ala318Thr	Coil2b	1	American [31]
7	p.Arg388His	Tail	1	American [31]
7	p.Arg377His	Tail	1	American [52]
8	p.Arg471His	Tail	1	American [31]
8	p.Tyr481^*∗*^	Tail	1	English [33]
10	p.Ser573Leu	Tail	1	American [52]
10	p.Arg541Ser	Tail	1	English [33]
11	p.Arg644Cys	Tail	2	German [26] and Chinese [58]
11	p.Arg654^*∗*^	Tail	1	American [31]

^*∗*^Stop codon.

## References

[1] Wu Z.-Y., Zhao G.-X., Chen W.-J. (2006). Mutation analysis of 218 Chinese patients with Wilson disease revealed no correlation between the canine copper toxicosis gene *MURR1* and Wilson disease. *Journal of Molecular Medicine*.

[2] James J., Zhang Y., Osinska H. (2000). Transgenic modeling of a cardiac troponin I mutation linked to familial hypertrophic cardiomyopathy. *Circulation Research*.

[3] Maron B. J., Doerer J. J., Haas T. S., Tierney D. M., Mueller F. O. (2009). Sudden deaths in young competitive athletes analysis of 1866 deaths in the United States, 1980–2006. *Circulation*.

[4] Gersh B. J., Maron B. J., Bonow R. O. (2011). 2011 ACCF/AHA guideline for the diagnosis and treatment of hypertrophic cardiomyopathy: a report of the American College of Cardiology Foundation/American Heart Association Task Force on practice guidelines developed in collaboration with the American Association for Thoracic Surgery, American Society of Echocardiography, American Society of Nuclear Cardiology, Heart Failure Society of America, Heart Rhythm Society, Society for Cardiovascular Angiography and Interventions, and Society of Thoracic Surgeons. *Journal of the American College of Cardiology*.

[5] Maron B. J. (2004). Hypertrophic cardiomyopathy: an important global disease. *The American Journal of Medicine*.

[6] Watkins H., Thierfelder L., Anan R. (1993). Independent origin of identical beta cardiac myosin heavy-chain mutations in hypertrophic cardiomyopathy. *The American Journal of Human Genetics*.

[7] Pinto Y. M., Wilde A. A. A. M., van Rijsingen I. A. W., Christiaans I., Deprez R. H. L., Elliott P. M. (2011). Clinical utility gene card for: hypertrophic cardiomyopathy (type 1–14). *European Journal of Human Genetics*.

[8] Kuster D. W. D., Sadayappan S. (2014). MYBPC3's alternate ending: consequences and therapeutic implications of a highly prevalent 25 bp deletion mutation. *Pflügers Archiv—European Journal of Physiology*.

[9] Colombo M. G., Botto N., Vittorini S., Paradossi U., Andreassi M. G. (2008). Clinical utility of genetic tests for inherited hypertrophic and dilated cardiomyopathies. *Cardiovascular Ultrasound*.

[10] Hershberger R. E., Hedges D. J., Morales A. (2013). Dilated cardiomyopathy: the complexity of a diverse genetic architecture. *Nature Reviews Cardiology*.

[11] Kubo T., Kitaoka H., Okawa M. (2011). Genetic screening and double mutation in Japanese patients with hypertrophic cardiomyopathy. *Circulation Journal*.

[12] Botto N., Vittorini S., Colombo M. G. (2010). A novel LMNA mutation (R189W) in familial dilated cardiomyopathy: Evidence for a ‘hot spot’ region at exon 3: a case report. *Cardiovascular Ultrasound*.

[13] Mestroni L., Maisch B., McKenna W. J. (1999). Guidelines for the study of familial dilated cardiomyopathies. *European Heart Journal*.

[14] Li H., Handsaker B., Wysoker A. (2009). The sequence alignment/map format and SAMtools. *Bioinformatics*.

[15] Wang K., Li M., Hakonarson H. (2010). ANNOVAR: functional annotation of genetic variants from high-throughput sequencing data. *Nucleic Acids Research*.

[16] Adzhubei I. A., Schmidt S., Peshkin L. (2010). A method and server for predicting damaging missense mutations. *Nature Methods*.

[17] Kumar P., Henikoff S., Ng P. C. (2009). Predicting the effects of coding non-synonymous variants on protein function using the SIFT algorithm. *Nature Protocols*.

[18] Schwarz J. M., Rödelsperger C., Schuelke M., Seelow D. (2010). MutationTaster evaluates disease-causing potential of sequence alterations. *Nature Methods*.

[19] Hirtle-Lewis M., Desbiens K., Ruel I. (2013). The genetics of dilated cardiomyopathy: a prioritized candidate gene study of LMNA, TNNT2, TCAP, and PLN. *Clinical Cardiology*.

[20] Sikkema-Raddatz B., Johansson L. F., Boer E. N. (2013). Targeted next-generation sequencing can replace Sanger sequencing in clinical diagnostics. *Human Mutation*.

[21] Sadayappan S., de Tombe P. P. (2014). Cardiac myosin binding protein-C as a central target of cardiac sarcomere signaling: a special mini review series. *European Journal of Physiology*.

[22] Sadayappan S., Finley N., Howarth J. W. (2008). Role of the acidic N′ region of cardiac troponin I in regulating myocardial function. *The FASEB Journal*.

[23] Wen Y., Pinto J. R., Gomes A. V. (2008). Functional consequences of the human cardiac troponin I hypertrophic cardiomyopathy mutation R145G in transgenic mice. *The Journal of Biological Chemistry*.

[24] Maron B. J., Niimura H., Casey S. A. (2001). Development of left ventricular hypertrophy in adults with hypertrophic cardiomyopathy caused by cardiac myosin-binding protein C gene mutations. *Journal of the American College of Cardiology*.

[25] Niimura H., Bachinski L. L., Sangwatanaroj S. (1998). Mutations in the gene for cardiac myosin-binding protein C and late-onset familial hypertrophic cardiomyopathy. *The New England Journal of Medicine*.

[26] Perrot A., Hussein S., Ruppert V. (2009). Identification of mutational hot spots in LMNA encoding lamin A/C in patients with familial dilated cardiomyopathy. *Basic Research in Cardiology*.

[27] Richard P., Charron P., Carrier L. (2003). Hypertrophic cardiomyopathy: distribution of disease genes, spectrum of mutations, and implications for a molecular diagnosis strategy. *Circulation*.

[28] Song K., Dubé M.-P., Lim J., Hwang I., Lee I., Kim J.-J. (2007). Lamin A/C mutations associated with familial and sporadic cases of dilated cardiomyopathy in Koreans. *Experimental and Molecular Medicine*.

[29] Hermida-Prieto M., Monserrat L., Castro-Beiras A. (2004). Familial dilated cardiomyopathy and isolated left ventricular noncompaction associated with lamin A/C gene mutations. *The American Journal of Cardiology*.

[30] Arbustini E., Pilotto A., Repetto A. (2002). Autosomal dominant dilated cardiomyopathy with atrioventricular block: a lamin A/C defect-related disease. *Journal of the American College of Cardiology*.

[31] Parks S. B., Kushner J. D., Nauman D. (2008). Lamin A/C mutation analysis in a cohort of 324 unrelated patients with idiopathic or familial dilated cardiomyopathy. *American Heart Journal*.

[32] Pethig K., Genschel J., Peters T. (2005). LMNA mutations in cardiac transplant recipients. *Cardiology*.

[33] Sylvius N., Bilinska Z. T., Veinot J. P. (2005). In vivo and in vitro examination of the functional significances of novel lamin gene mutations in heart failure patients. *Journal of Medical Genetics*.

[34] Kärkkäinen S., Reisseil E., Heliö T. (2006). Novel mutations in the lamin A/C gene in heart transplant recipients with end stage dilated cardiomyopathy. *Heart*.

[35] Berge K. E., Leren T. P. (2014). Genetics of hypertrophic cardiomyopathy in Norway. *Clinical Genetics*.

[36] Frisso G., Limongelli G., Pacileo G. (2009). A child cohort study from southern Italy enlarges the genetic spectrum of hypertrophic cardiomyopathy. *Clinical Genetics*.

[37] Arad M., Penas-Lado M., Monserrat L. (2005). Gene mutations in apical hypertrophic cardiomyopathy. *Circulation*.

[38] Zou Y., Wang J., Liu X. (2013). Multiple gene mutations, not the type of mutation, are the modifier of left ventricle hypertrophy in patients with hypertrophic cardiomyopathy. *Molecular Biology Reports*.

[39] Carballo S., Robinson P., Otway R. (2009). Identification and functional characterization of cardiac troponin i as a novel disease gene in autosomal dominant dilated cardiomyopathy. *Circulation Research*.

[40] Frazier A., Judge D. P., Schulman S. P., Johnson N., Holmes K. W., Murphy A. M. (2008). Familial hypertrophic cardiomyopathy associated with cardiac *β*-myosin heavy chain and troponin I mutations. *Pediatric Cardiology*.

[41] Van Driest S. L., Ellsworth E. G., Ommen S. R., Tajik A. J., Gersh B. J., Ackerman M. J. (2003). Prevalence and spectrum of thin filament mutations in an outpatient referral population with hypertrophic cardiomyopathy. *Circulation*.

[42] Choi J.-O., Yu C.-W., Nah J. C. (2010). Long-term outcome of 4 Korean families with hypertrophic cardiomyopathy caused by 4 different mutations. *Clinical Cardiology*.

[43] Mogensen J., Murphy R. T., Kubo T. (2004). Frequency and clinical expression of cardiac troponin I mutations in 748 consecutive families with hypertrophic cardiomyopathy. *Journal of the American College of Cardiology*.

[44] Kimura A., Harada H., Park J.-E. (1997). Mutations in the cardiac troponin I gene associated with hypertrophic cardiomyopathy. *Nature Genetics*.

[45] Bonne G., Mercuri E., Muchir A. (2000). Clinical and molecular genetic spectrum of autosomal dominant Emery-Dreifuss muscular dystrophy due to mutations of the lamin A/C gene. *Annals of Neurology*.

[46] Moon J. C., Mogensen J., Elliott P. M. (2005). Myocardial late gadolinium enhancement cardiovascular magnetic resonance in hypertrophic cardiomyopathy caused by mutations in troponin I. *Heart*.

[47] Kaski J. P., Syrris P., Esteban M. T. T. (2009). Prevalence of sarcomere protein gene mutations in preadolescent children with hypertrophic cardiomyopathy. *Circulation: Cardiovascular Genetics*.

[48] Erdmann J., Daehmlow S., Wischke S. (2003). Mutation spectrum in a large cohort of unrelated consecutive patients with hypertrophic cardiomyopathy. *Clinical Genetics*.

[49] van den Wijngaard A., Volders P., van Tintelen J. P. (2011). Recurrent and founder mutations in the Netherlands: cardiac troponin I (TNNI3) gene mutations as a cause of severe forms of hypertrophic and restrictive cardiomyopathy. *Netherlands Heart Journal*.

[50] Niimura H., Patton K. K., McKenna W. J. (2002). Sarcomere protein gene mutations in hypertrophic cardiomyopathy of the elderly. *Circulation*.

[51] Gomes A. V., Potter J. D. (2004). Cellular and molecular aspects of familial hypertrophic cardiomyopathy caused by mutations in the cardiac troponin I gene. *Molecular and Cellular Biochemistry*.

[52] Taylor M. R. G., Fain P. R., Sinagra G. (2003). Natural history of dilated cardiomyopathy due to lamin A/C gene mutations. *Journal of the American College of Cardiology*.

[53] Fatkin D., Macrae C., Sasaki T. (1999). Missense mutations in the rod domain of the lamin A/C gene as causes of dilated cardiomyopathy and conduction-system disease. *The New England Journal of Medicine*.

[54] Wang H., Wang J., Zheng W. (2006). Mutation Glu82Lys in lamin A/C gene is associated with cardiomyopathy and conduction defect. *Biochemical and Biophysical Research Communications*.

[55] van Tintelen J. P., Hofstra R. M. W., Katerberg H. (2007). High yield of LMNA mutations in patients with dilated cardiomyopathy and/or conduction disease referred to cardiogenetics outpatient clinics. *The American Heart Journal*.

[56] Lai C.-C., Yeh Y.-H., Hsieh W.-P. (2013). Whole-exome sequencing to identify a novel LMNA gene mutation associated with inherited cardiac conduction disease. *PLoS ONE*.

[57] Roncarati R., Viviani Anselmi C., Krawitz P. (2013). Doubly heterozygous LMNA and TTN mutations revealed by exome sequencing in a severe form of dilated cardiomyopathy. *European Journal of Human Genetics*.

[58] Sun L.-P., Wang L., Wang H., Zhang Y.-H., Pu J.-L. (2010). Connexin 43 remodeling induced by LMNA gene mutation Glu82Lys in familial dilated cardiomyopathy with atrial ventricular block. *Chinese Medical Journal*.

